# Transmural heterogeneity of microstructural remodeling in pacing induced heart failure measured by diffusion tensor MRI

**DOI:** 10.1186/1532-429X-15-S1-P119

**Published:** 2013-01-30

**Authors:** Geoffrey L Kung, Sarah Ouadah, Yu-Cheng Hsieh, Alan Garfinkel, Peng-Sheng Chen, Daniel Ennis

**Affiliations:** 1 Department of Radiological Sciences, University of California, Los Angeles, Los Angeles, CA, USA; 2 Department of Bioengineering, University of California, Los Angeles, Los Angeles, CA, USA; 3 Krannert Institute of Cardiology and the Division of Cardiology, Indiana University School of Medicine, Indianapolis, IN, USA; 4 Department of Medicine (Cardiology), University of California, Los Angeles, Los Angeles, CA, USA

## Background

Diffusion tensor magnetic resonance imaging (DT-MRI) enables 3D evaluation of whole heart microstructure. DT invariants evaluate microstructural remodeling by quantifying trace (increases with decreasing cellularity), fractional anisotropy (FA, decreases with increasing fibrosis), and tissue mode (decreases with increasing fiber disarray) [1]. We have shown that DT invariant data identifies significant global microstructural remodeling (increase in trace and decrease in FA) in the pacing induced heart failure (HF) model [2]. The objective of this study was to quantify transmural microstructural remodeling between normal and HF myocardium using DT invariants.

## Methods

HF was induced in 10-12 month old New Zealand White female rabbits (N=8) with an epicardial pacing lead placed in the lateral LV wall and tachycardia pacing at 250 beats per minute (bpm) for 3 days, 300 bpm for 3 days, and 350 bpm for 3-4 weeks. Normal weight matched rabbits (N=5) served as controls (CNTL). Hearts were excised, formalin fixed, and DT-MRI was performed on a 7T scanner (Bruker, Billerica, MA) (24 diffusion gradient directions, 6 nulls, TE/TR=30/500 ms, b-value=1000 s/mm^2^, 0.5 x 0.5 x 0.75 mm resolution). Trace, FA, and mode were segmented into epicardial, midwall, and endocardial regions. Bootstrapped histograms with 95% confidence intervals (95%-CIs) of the de-correlated (via decimation by the auto-correlation length) and segmented invariant data were defined to make statistical comparisons of non-Gaussian datasets tractable. Two-group comparisons of median invariant data of each heart were used to test for significant differences (p < 0.05) between HF and CNTL in each transmural region.

## Results

**Figure 1:**
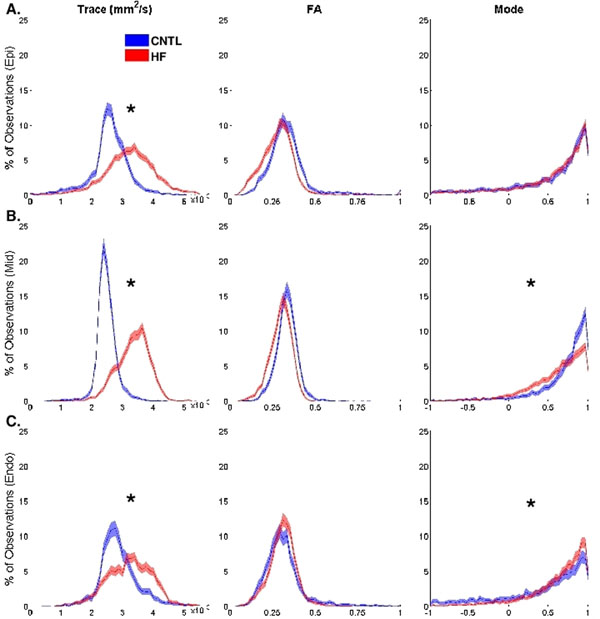
Bootstrapped histograms with 95%-CIs of HF (blue) and CNTL (red) groups using de-correlated data for trace, FA, and mode in A) epicardial, B) midwall, and C) endocardial regions. Invariant histograms labeled with "*" signify a statistically significant shift in median value from CNTL to HF. An increase in trace is significant in all transmural regions (all p < 0.04) in HF compared to CNTL. A decrease in mode is significant (p = 0.04) in midwall HF compared to midwall CNTL. An increase in mode is significant (p = 0.04) in endocardial HF compared to endocardial CNTL.

Figure 1 depicts bootstrapped histograms with 95%-CIs for transmurally segmented invariant data across groups (HF vs CNTL). Trace significantly increased from CNTL to HF in all transmural regions (all p < 0.04). An increase in trace implies a decrease in diffusive barriers per voxel or decreased cellularity [3]. FA differences from CNTL to HF were insignificant in all regions. Increased myocyte size without significant changes in fibrosis have been histologically observed in the pacing induced HF model [4], which is consistent with an increase in trace without a change in FA. Mode significantly decreased in midwall and increased in endocardium from CNTL to HF (both p = 0.04) but did not significantly shift in epicardium. A decrease in mode implies fiber disarray as local diffusion shifts towards planar anisotropy. An increase in mode implies a loss of sheet structure as local diffusion shifts towards linear anisotropy.

## Conclusions

DT invariant data indentify statistically significant microstructural remodeling in the pacing induced HF model within epicardial, midwall, and endocardial regions.

## Funding

Kung - AHA Grant 12PRE9160024, Chen - NIH Grants P01HL78931, R01HL78932, a Medtronic-Zipes Endowment and the Indiana University-Indiana School of Medicine Strategic Research Initiative.
